# Evidence for Complex Formation of the *Bacillus cereus* Haemolysin BL Components in Solution

**DOI:** 10.3390/toxins9090288

**Published:** 2017-09-16

**Authors:** Franziska Tausch, Richard Dietrich, Kristina Schauer, Robert Janowski, Dierk Niessing, Erwin Märtlbauer, Nadja Jessberger

**Affiliations:** 1 Department of Veterinary Sciences, Faculty of Veterinary Medicine, Ludwig-Maximilians-Universität München, Schönleutnerstr 8, 85764 Oberschleißheim, Germany; Franziska.Tausch@gmx.de (F.T.); r.dietrich@mh.vetmed.uni-muenchen.de (R.D.); kristina.schauer@mh.vetmed.uni-muenchen.de (K.S.); e.maertlbauer@mh.vetmed.uni-muenchen.de (E.M.); 2 Institute of Structural Biology, Helmholtz Zentrum München-German Research Center for Environmental Health, Ingolstädter Landstr. 1, 85764 Neuherberg, Germany; robert.janowski@helmholtz-muenchen.de (R.J.); niessing@helmholtz-muenchen.de (D.N.); 3 Biomedical Center of the Ludwig-Maximilians-Universität München, Department of Cell Biology, 82152 Planegg-Martinsried, Germany

**Keywords:** *Bacillus cereus*, complex formation, enterotoxins, haemolysin BL, monoclonal antibodies

## Abstract

Haemolysin BL is an important virulence factor regarding the diarrheal type of food poisoning caused by *Bacillus cereus*. However, the pathogenic importance of this three-component enterotoxin is difficult to access, as nearly all natural *B. cereus* culture supernatants additionally contain the highly cytotoxic Nhe, the second three-component toxin involved in the aetiology of *B. cereus*-induced food-borne diseases. To better address the toxic properties of the Hbl complex, a system for overexpression and purification of functional, cytotoxic, recombinant (r)Hbl components L_2_, L_1_ and B from *E. coli* was established and an *nheABC* deletion mutant was constructed from *B. cereus* reference strain F837/76. Furthermore, 35 hybridoma cell lines producing monoclonal antibodies (mAbs) against Hbl L_2_, L_1_ and B were generated. While mAbs 1H9 and 1D8 neutralized Hbl toxicity and thus, represent important tools for future investigations of the mode-of-action of Hbl on the target cell surface, mAb 1D7, in contrast, even enhanced Hbl toxicity by supporting the binding of Hbl B to the cell surface. By using the specific mAbs in Dot blots, indirect and hybrid sandwich enzyme immuno assays (EIAs), complex formation between Hbl L_1_ and B, as well as L_1_ and L_2_ in solution could be shown for the first time. Surface plasmon resonance experiments with the rHbl components confirmed these results with K_D_ values of 4.7 × 10^−7^ M and 1.5 × 10^−7^ M, respectively. These findings together with the newly created tools lay the foundation for the detailed elucidation of the molecular mode-of-action of the highly complex three-component Hbl toxin.

## 1. Introduction

The Gram-positive, facultative anaerobe and rod-shaped bacterium *Bacillus cereus* has become increasingly important as a cause of food poisoning outbreaks. It is ubiquitous in soil, sediments, dust and plants [1]. From there, it can easily be spread to different foods. *B. cereus* is found in an extraordinary variety of food, as for example in milk and dairy products, rice and pasta or spices, dry foods and vegetables [1,2,3]. Food poisoning caused by *B. cereus* is mostly moderate and self-limiting, but also severe and even lethal cases have been reported [4,5,6]. 

*B. cereus* causes mainly two types of foodborne diseases, an emetic and a diarrheal form. The first is characterized by nausea and vomiting and is caused by the cyclic dodecadepsipeptide cereulid, which is produced in foods before consumption [7,8]. Three types of enterotoxins are responsible for the diarrheal form. These are produced foremost in the intestine from viable bacteria that, most likely as spores, survived the stomach passage [9]. These enterotoxins are the three component complexes Hbl (haemolysin BL; [10]) and Nhe (non-haemolytic enterotoxin; [11]), and the single protein CytK (cytotoxin K; [4]). CytK has been reported to be haemolytic, cytotoxic, necrotic and is a member of the family of β-barrel pore forming toxins [12,13]. Only very few strains express the highly toxic CytK1 variant and are classified as *Bacillus cytotoxicus* [14]. 

The more complex Nhe and Hbl toxins consist of three components each, namely NheA, B and C and Hbl L_2_, L_1_ and B. It has been shown that for both toxins all three components are needed for maximum biological activity [10,15]. Intensive studies have been performed on the mode of action of Nhe. It has been shown that NheB and C, but not NheA can bind to Vero cells. Nhe is a pore forming toxin inducing cell lysis with an optimum molar ratio for maximum toxicity of A:B:C = 10:10:1. Increasing ratios of NheC lead to inhibition of the toxic activity [15,16]. Moreover, a specific binding order, i.e., NheC-B-A, is necessary for Nhe activity [17,18,19]. The mode-of-action of Nhe was clarified with the help of monoclonal antibodies (mAbs), which hinder the interaction between the single Nhe components and thus, neutralize the toxic activity [18,20]. On the other hand, these specific mAbs were also used to detect complex formation of Nhe components in culture supernatants, such as the interaction between NheB and C [21].

Hbl was originally purified from *B. cereus* strain F837/76 and a binding component (B) as well as two lytic components (L_2_ and L_1_) were identified [22,23]. Several different ideas about the mode of action of Hbl exist. First, it has been suggested that each component is able to bind individually to erythrocytes and thus, that they assemble into a “membrane attack complex”, then form a transmembrane pore and lyse the cells [24]. Osmotic protection assays showed that Hbl is a pore forming toxin and that pores are smaller than 1.2 nm [24]. On blood agar plates, it was observed that excess of Hbl L_1_ or B inhibits haemolytic activity [24,25]. Thus, a certain concentration ratio of the Hbl components might also be required for maximum activity, but this is, in contrast to Nhe, so far unknown. In another approach it was shown that sequential binding in the specific binding order of Hbl B-L_1_-L_2_ leads to toxic activity in cell viability tests on Chinese hamster ovary cells [19]. As the crystal structure of Hbl B was solved, a high structural similarity to *E. coli* haemolysin E (HlyE; ClyA) was observed despite low sequence homology [26]. Based on this similarity, another model was proposed in which Hbl B alone might be able to oligomerize on the cell surface and form a pore. L_2_ and L_1_, which are definitely required for toxic activity, might either stabilize B, induce conformational changes to B or even enter the cell [26]. 

Investigating Hbl activity in natural *B. cereus* culture supernatants is difficult, as all known enteropathogenic strains that bear *hbl* also have the *nhe* operon. The *nhe* genes can be found in all enteropathogenic *B. cereus* strains, the *hbl* genes in about 45%–65% [27,28,29]. Early studies on Hbl activity were carried out with proteins purified via anion exchange chromatography from *B. cereus* culture supernatants [23,30]. These might have contained trace contaminations and the use of recombinantly expressed and purified Hbl (rHbl) components was recommended [10]. For a long time rHbl components could not be generated, it was even suggested that the individual components might be toxic for *E. coli* [31]. Only in 2013, Sastalla and co-workers were able to overexpress *B. cereus* Nhe and Hbl proteins in a *Bacillus anthracis* expression system [19]. 

The aim of this study was to create suitable and effective tools by which the complex mode-of-action of Hbl can be investigated. First, functional rHbl components were overexpressed and purified from *E. coli* in an easy, fast and secure (S1) system. Secondly, a deletion mutant from *B. cereus* reference strain F837/76 was constructed lacking the *nhe* operon. Finally, a whole set of highly specific mAbs against the three Hbl components was generated. Based on these reagents, it could be shown for the first time that Hbl components L_1_ and B, as well as L_1_ and L_2_ form complexes in solution.

## 2. Results

### 2.1. Generation of Functional Recombinant Hbl (rHbl) Components and a *Δ*nheABC Mutant

**Figure 1:**
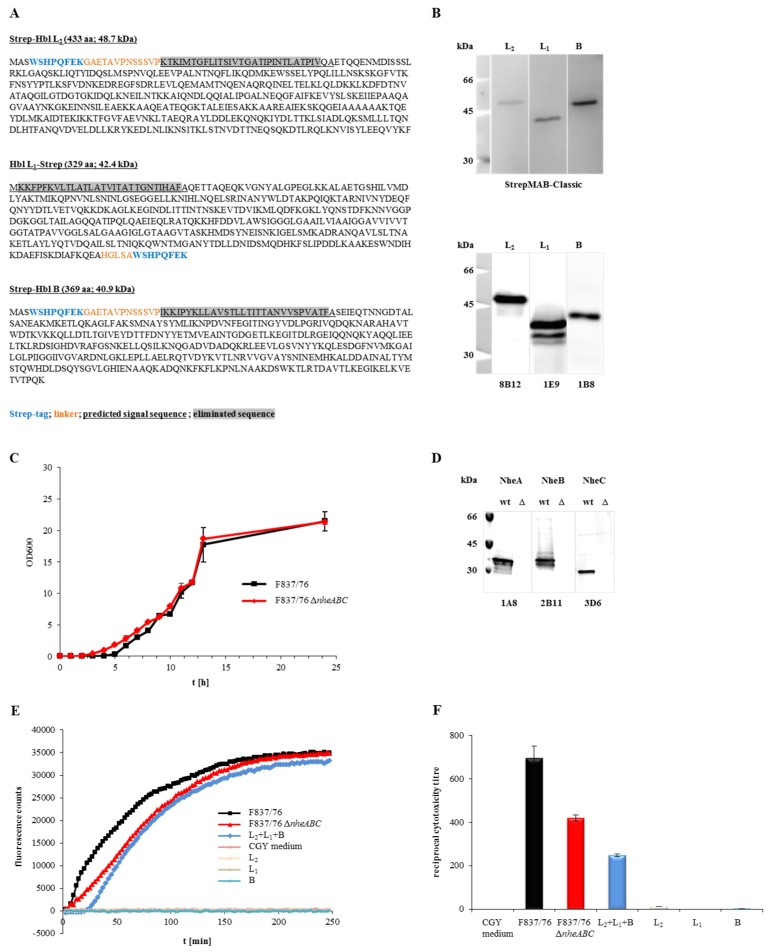
Properties of the recombinant Hbl components and the *nheABC* deletion strain. (**A**) Amino acid sequences of rHbl L_2_, L_1_ and B. Strep-tags are shown in blue, linker amino acids in orange letters, the predicted secretion signal sequences are underlined and eliminated sequences are highlighted in grey. From these sequences the molecular weight was predicted (http://web.expasy.org/compute_pi/). (**B**) Detection of the rHbl components in Western blots. The strep-specific StrepMAB-Classic was used for detection (upper blot), as well as the Hbl-specific mAbs 8B12 (L_2_) [32], 1E9 (L_1_) [29] and 1B8 [29] (lower blot). (**C**) Growth of *B. cereus* strains F837/76 and F837/76 ∆*nheABC* in LB medium. Medium was inoculated to an OD_600_ of 0.005 and growth at 37 °C was monitored for 24 h. (**D**) Western blot. NheA, B and C were detected in the supernatant of strain F837/76 using the mAbs 1A8 [20], 2B11 [20] and 3D6 [21], respectively. These proteins were not detected in the supernatant of the *nheABC* deletion mutant. (**E**) Influx of PI into Vero cells, represented by increasing fluorescence counts. 1.5 pmol/μL rHbl components were either used separately or mixed in a 1:1:1 ratio and applied in 1:40 dilution to the cells, as was supernatant of F837/76 ∆*nheABC*. CGY medium and supernatant of F837/76 were used as controls. (**F**) Results of a WST-1-bioassay (Vero cells) determining toxic activity of the *nheABC* deletion strain and the rHbl components (1.5 pmol/μL each, separately or mixed in a 1:1:1 ratio). CGY medium and supernatant of F837/76 were used as controls.

To generate functional, recombinant Hbl toxin, an approach based on the Strep-tag^®^ purification system (iba lifesciences) was used. rHbl L_2_ and B were expressed with an *N*-terminal, rHbl L_1_ with a C-terminal strep-tag. Sequences encoding putative secretion signal peptides were eliminated. Amino acid sequences and predicted molecular weight of the new recombinant proteins are shown in Figure 1A. The proteins were overexpressed in *E. coli* BL21 (DE3) and purified via affinity chromatography. Purification was controlled in Western blots (Figure 1B). Each of the single components was detected by a strep-specific antibody as well as by the Hbl-specific mAbs. rHbl L_2_ and L_1_ were detected at their predicted molecular weight of 48.7 and 42.4 kDa, while rHbl B appeared slightly bigger in Western blot analyses than its predicted 40.9 kDa. For rHbl L_1_, a second band was detected with the L_1_- but not with the strep-specific mAb, indicating partial cleavage of the tag. 

A ∆*nheABC* mutant was constructed from *B. cereus* strain F837/76. The *nhe* operon was replaced by a spectinomycin resistance cassette, which was confirmed by sequencing. The start and stop codon, as well as the first 36 and the last 18 bp of the operon remained. No phenotypical differences could be detected compared to the wild type (Figure 1C). Western blots (Figure 1D) confirmed that no Nhe toxin was produced by the deletion strain. 

To determine the toxic activity of the newly generated rHbl components and the ∆*nheABC* strain, they were analysed by PI influx tests as well as by WST-1-bioassays. The rHbl components were clearly able to induce pore formation in Vero cells when applied simultaneously (Figure 1E). Fluorescence could be measured after approximately 25 min, while supernatant of strain F837/76, which was used as control, caused almost immediate PI influx. Simultaneously applied rHbl components were also able to kill Vero cells (Figure 1F), although the reciprocal titre (the dilution to get 50% viable cells after 24 h) was significantly lower than that of F837/76 supernatant (250 compared to 700). The ∆*nheABC* mutant showed decelerated PI influx compared to the wild type (Figure 1E) as well as a reduced reciprocal cytotoxicity titre (400 compared to 700; Figure 1F). Altogether, these results prove that, in contrary to earlier claims, overexpression and purification of functional rHbl in *E. coli* as well as the deletion of the *nhe* operon in *B. cereus* is indeed possible.

### 2.2. Generation of Monoclonal Antibodies (mAbs) against Hbl

Highly specific mAbs were generated in this study according to established procedures [20,32]. For immunogen preparation, strain MHI 1532 was chosen, as it showed the highest Hbl B titres in indirect EIAs and the highest Hbl L_2_ titres in sandwich EIAs of all strains tested in preliminary experiments (data not shown). The immunogen was gained by purification of the culture supernatant via Hbl B-specific IAC with the already established Hbl B-specific mAb 1B8 [29]. Purified Hbl toxin was used for immunization and two booster injections of mice. After cell fusion, 35 hybridoma cell lines secreting Hbl-reactive antibodies could be identified (Table S1). The target antigens of the mAbs were determined in indirect EIAs using rHbl components. Twenty-nine hybridoma cell lines produced Hbl B-specific mAbs, four mAbs were cross-reactive with Hbl B and Hbl L_1_, and two mAbs were specific for Hbl L_2_. As mice were immunized with a preparation of IAC-purified Hbl B from *B. cereus* culture supernatants (see above), this finding gave a first hint that the single Hbl components form complexes in solution.

With respect to affinity, stability and productivity of the hybridoma cell lines, 1G8 and 12D12 (Hbl B/L_1_), 1D8 and 1H9 (Hbl L_2_), and 2G4, 1D7, 1D12 and 1C12 (Hbl B) were chosen for mass production and the respective, purified mAbs were used for further experiments.

### 2.3. The Generated mAbs Show Neutralizing and Enhancing Properties towards Hbl Toxicity

**Figure 2:**
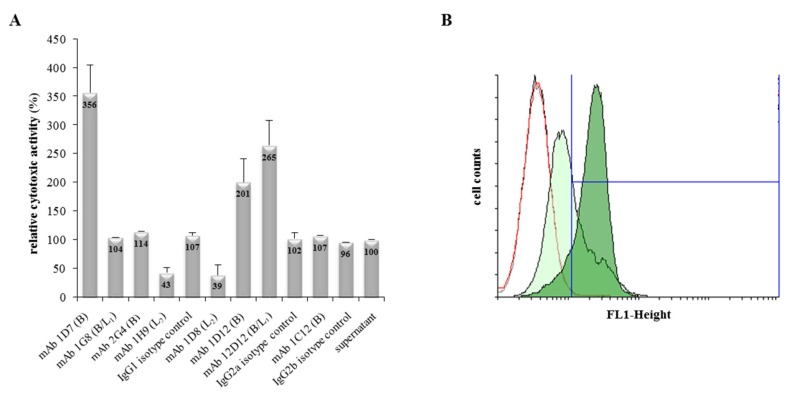
Influence of mAbs on Hbl toxicity. (**A**) WST-1-Bioassay on Vero cells. Supernatant of F837/76 ∆*nheABC* was applied as serial dilution. mAb (10 μg/well) was added to each dilution and incubated with the cells for 24 h. Cytotoxicity titres were determined by addition of WST-1. The reciprocal titre of the untreated *B. cereus* supernatant was set to 100%. (**B**) Flow cytometry results of Vero cells treated with rHbl B. Cells were incubated for 1 h with either only buffer (black curve), Hbl B-specific mAb 1D7 (red curve), rHbl B (light green filled) or rHbl B + mAb 1D7 (dark green filled). Cell-bound rHbl B was detected by using Alexa Fluor^®^ 488-labelled mAb 1G8 (Hbl B-specific).

To investigate the neutralizing properties of the generated mAbs against Hbl, antibodies were applied simultaneously with culture supernatants of *B. cereus* strain F837/76 ∆*nheABC* in WST-1-bioassays on Vero cells. The reciprocal titre of the untreated supernatant was set to 100% and relative cytotoxicity of all tested samples was compared to that value. mAbs 1H9 and 1D8 (Hbl L_2_-specific) clearly reduced the toxic activity of the Hbl-containing *B. cereus* supernatant by approximately 60%, while unexpectedly mAbs 1D7 (Hbl B), 1D12 (Hbl B) and 12D12 (Hbl B/L_1_) enhanced it by 256, 101 and 165%, respectively (Figure 2A). Isotype controls as well as all other tested mAbs showed no significant influence on Hbl toxicity. The three mAbs enhancing Hbl toxicity were used in flow cytometric analyses to determine their influence on Hbl B binding to Vero cells. For that, Vero cells were incubated for 1 h with rHbl B (6.5 pmol/mL) and then probed with Alexa Fluor^®^ 488-labelled mAb 1G8 (Hbl B/L_1_-specific). These settings resulted in approximately 42% fluorescence (FL1)-positive cells (Table 1). Co-incubation of rHbl B and mAbs 1D12 and 12D12 on Vero cells resulted in no and only slightly enhanced number of FL1-positive cells, respectively. However, when rHbl B was co-incubated on Vero cells with the Hbl B-reactive mAb 1D7, nearly all cells were FL1 positive (96.37%) (Table 1 and Figure 2B). Under consecutive incubation conditions, in which Vero cells were first incubated with rHbl B and then, after washing, with mAb 1D7, this effect was not seen. Negative and isotype controls proved the specificity of the reaction (Table 1). Overall, it could be demonstrated that rHbl B binds specifically to Vero cells and that Hbl B-specific mAb 1D7 enhances this binding by a so far unknown mechanism. The variety of enhancing or neutralizing properties indirectly indicates that the generated mAbs recognize different epitopes.

### 2.4. Complex Formation of rHbl Components

**Figure 3:**
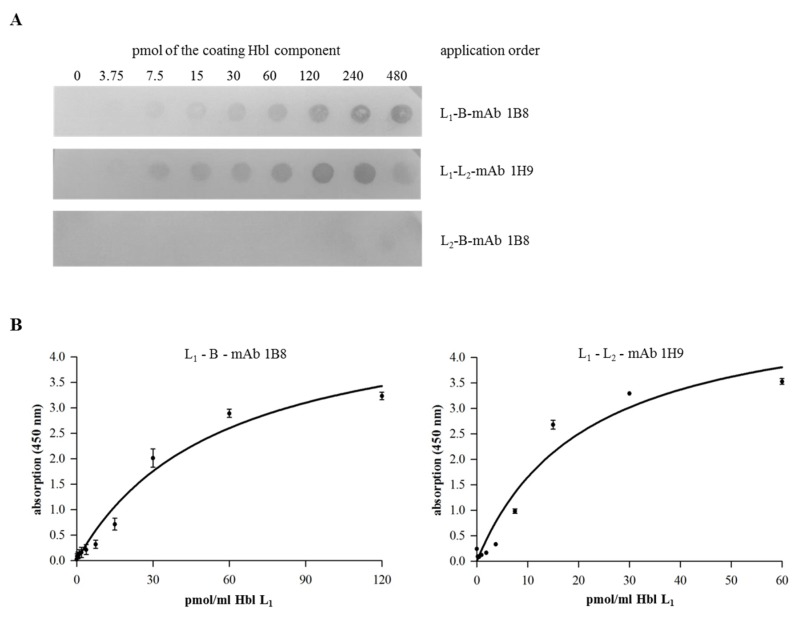
Detection of rHbl complex formation. (**A**) Dot blot. PVDF membranes were coated with rising concentrations (3.75–480 pmol) of different rHbl components. After blocking, the membrane was incubated in PBS with the second component (30 pmol). Proteins were detected using the Hbl B-specific mAb 1B8 [29] and the Hbl L_2_-specific mAb 1H9 (this study). Inversion of the protein order showed similar results and negative controls confirmed the specificity of the reaction (see Figure S1). (**B**) Indirect EIA. The first rHbl component was applied as serial dilution to a microtiter plate. After washing, the second rHbl component was applied in constant concentration (60 pmol/mL). After blocking, Hbl B-specific mAb 1B8 [29] and Hbl L_2_-specific mAb 1H9 (this study) were applied, respectively, followed by rabbit-anti-mouse-HRP conjugate for detection. Details on the non-linear regression are shown in Table S2.

**Figure 4:**
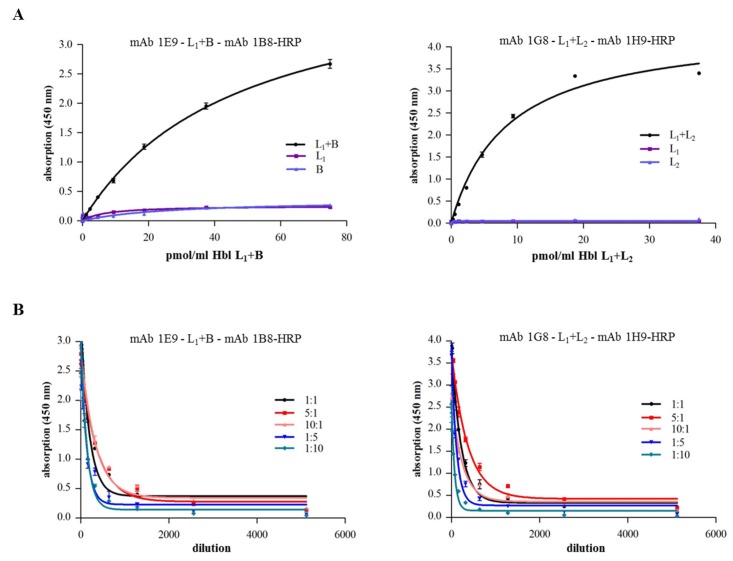
Detection of rHbl complex formation via sandwich EIAs. (**A**) Microtiter plates were coated with mAbs 1E9 (Hbl L_1_) [29] and 1G8 (Hbl B/L_1_) (this study), respectively. rHbl components were pre-mixed (each 1.5 pmol/μL in an 1:1 ratio), incubated for 30 min at RT and, after blocking, applied as serial dilution. After washing, the specific conjugates 1B8-HRP (Hbl B) [29] and 1H9-HRP (Hbl L_2_) (this study) were used for detection. (**B**) In an analogous approach, rHbl components were pre-mixed in different concentration ratios (each 1.5 pmol/μL, ratios 1:1, 5:1, 10:1, 1:5 and 1:10). Details on the non-linear regression are shown in Table S2.

The panel of Hbl-specific mAbs with putative different epitope-specificity offered the opportunity to investigate possible interactions between the single rHbl components. For that, Dot blots and enzyme immunoassays were performed. For Dot blot analyses, a dilution series (480–3.75 pmol) of the first rHbl component was applied to a PVDF membrane, which was then incubated with the second rHbl component (30 pmol in PBS). For detection, a specific mAb against the second rHbl component was used. Hereby, specific interactions between rHbl L_1_ and B as well as rHbl L_1_ and L_2_, but not rHbl L_2_ and B were demonstrated, whereat the application order of the rHbl components was not important (Figure 3A and Figure S1). A similar composition applied in indirect EIAs confirmed the specific complex formation between rHbl L_1_ and B, and rHbl L_1_ and L_2_ (Figure 3B). These complexes could also be detected in a different approach where rHbl L_1_ and B, and rHbl L_1_ and L_2_ were pre-incubated for 30 min and subsequently detected in highly specific sandwich EIAs (Figure 4A). This variant also allowed the comparison of different ratios of the single rHbl components (Figure 4B). Hbl complexes were detected in all tested variants. In the 1E9-1B8-sandwich EIA, rHbl L_1_ + B complexes were less well detected with excess of B. Excess of L_1_ had little influence on complex detection compared to the 1:1 ratio. In the 1G8 (Hbl HblB/L_1_-1H9 Hbl L_2_)-sandwich EIA, rHbl L_1_+L_2_ complexes were less well detected with excess of L_2_. A ratio of L_1_:L_2_ of 5:1 resulted in the highest absorption values, while further excess (10:1) showed no difference to the 1:1 control. Complex formation between rHbl L_2_ and B could be detected neither in indirect nor in sandwich EIAs (data not shown). 

**Figure 5:**
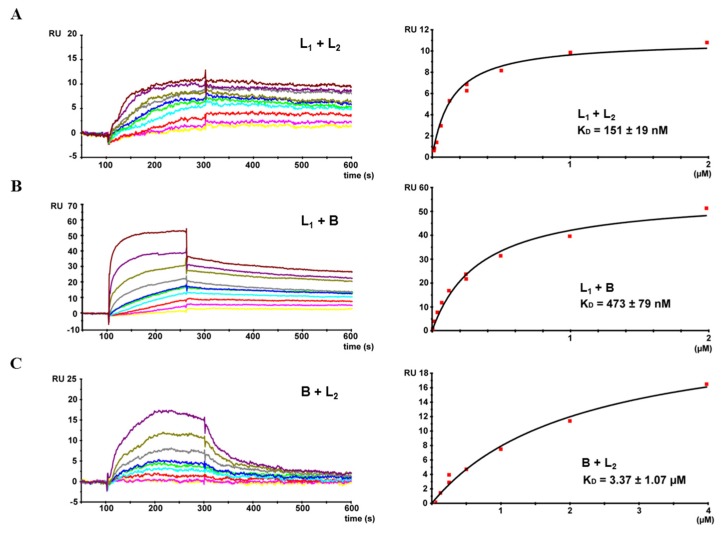
SPR measurement of the rHbl components. On the left side, a representative sensogram is depicted. Right panels show binding curves fitted to a one-site-binding model with calculated equilibrium dissociation rates (K_D_) and errors as standard deviations from three or four independent experiments. (**A**) rHbl L_1_ immobilized on a sensor chip. Concentration series (7.8 nM—yellow, 15.6 nM—magenta, 31.2 nM—red, 62.5 nM—cyan, 125 nM—green, 250 nM—blue and gray, 500 nM—dark yellow, 1 μM—dark magenta and 2 μM—dark red) of rHbl L_2_ were applied. (**B**) rHbl L_1_ immobilized on a sensor chip. Concentration series (7.8 nM—yellow, 15.6 nM—magenta, 31.2 nM—red, 62.5 nM—cyan, 125 nM—green, 250 nM—blue and gray, 500 nM—dark yellow, 1 μM—dark magenta and 2 μM—dark red) of rHbl B were applied. (**C**) rHbl B immobilized on a sensor chip. Concentration series (31.2 nM—yellow, 62.5 nM—magenta, 125 nM—red, 250 nM—cyan and green, 500 nM—blue, 1 μM—gray, 2 μM—dark yellow and 4 μM—dark magenta) of rHbl L2 were applied. RU: response units. Details on the non-linear regression are shown in Table S2.

Finally, complex formation between rHbl L_1_ and L_2_ as well as rHbl L_1_ and B was confirmed via surface plasmon resonance (SPR). For that, rHbl L_1_ was immobilized, and rising concentrations (from 7.8 nM to 2 μM) of rHbl L_2_ and B were added. K_D_ values of 1.5 × 10^−7^ M and 4.7 × 10^−7^ M were determined, respectively (Figure 5A,B). When rHbl L_2_ was coupled to the sensor chip, only interaction with rHbl L_1_ was detected, not with B (data not shown). Interestingly, switching rHbl B and L_2_ (B coupled and L_2_ used as ligand), resulted in specific interaction of the two proteins, with a, compared to the results obtained in Figure 5A,B, relative low K_D_ value of 3.4 × 10^−6^ M (Figure 5C).

### 2.5. Complex Formation in Natural B. cereus Culture Supernatants

**Figure 6:**
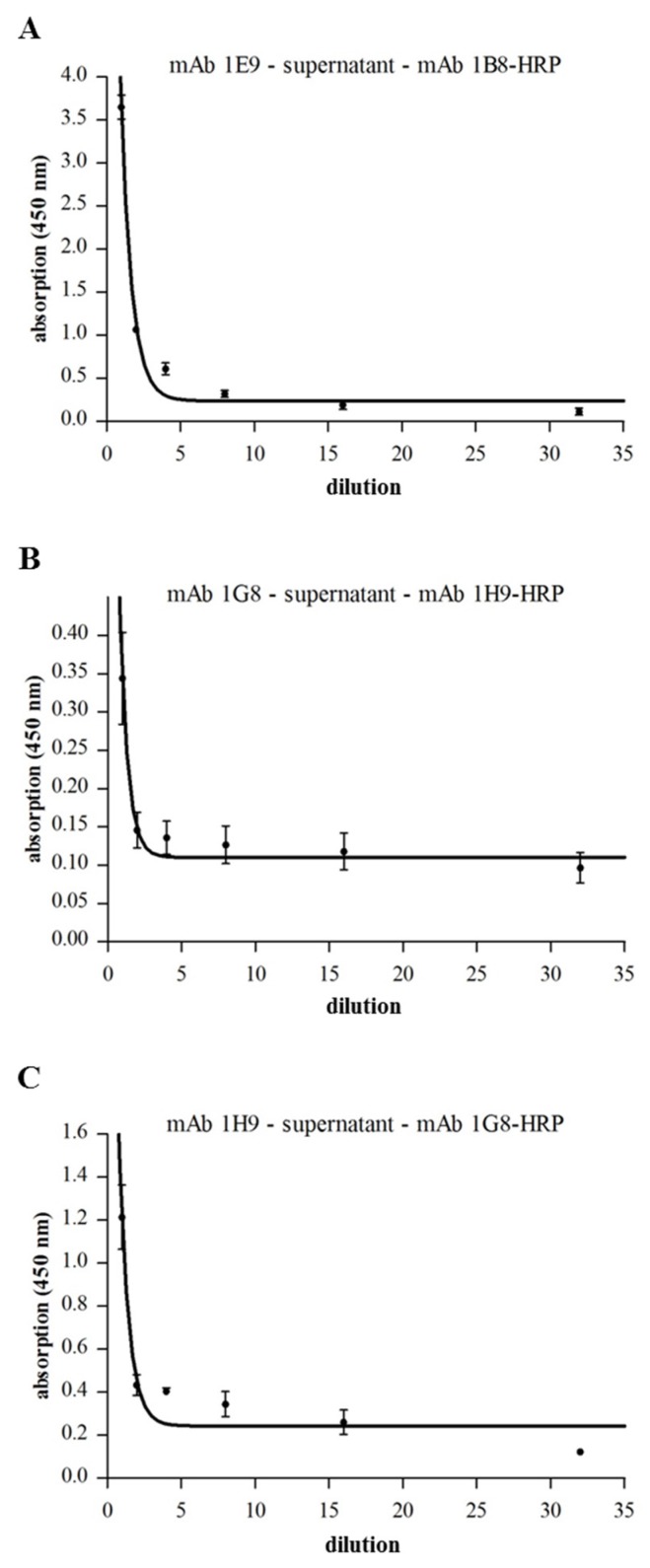
Hbl complex formation in natural *B. cereus* supernatants. In all tests, supernatant of strain F837/76 was applied as serial dilution. (**A**) Sandwich EIA specific for Hbl L_1_-B complexes, using mAb 1E9 (Hbl L_1_) and the 1B8-HRP conjugate (Hbl B) [29]. (**B**) Sandwich EIA specific for Hbl L_1_-L_2_ complexes, using mAb 1G8 (Hbl L_1_) and the 1H9-HRP conjugate (Hbl L_2_) (this study). (**C**) Due to weak results in Figure 6B, the capture and detection mAbs for Hbl L_1_ (1G8) and L_2_ (1H9) were switched. Details on the non-linear regression are shown in Table S2.

To detect complex formation of Hbl components in natural *B. cereus* supernatants, sandwich EIAs were performed with serial dilutions of F837/76 supernatant (analogously to Figure 4). Hbl L_1_-B complexes could be detected (Figure 6A). As only very weak signals were detected in the Hbl L_1_-L_2_-specific sandwich EIA (Figure 6B), the specific mAbs were switched (anti-L_2_ 1H9 as capture and anti-L_1_ 1G8 as detection antibody) and clearer signals were determined (Figure 6C). Altogether, with the complex sandwich EIAs, effective tools were developed to prove Hbl complex formation not only within recombinant Hbl components, but also in natural *B. cereus* culture supernatants.

**Figure 7:**
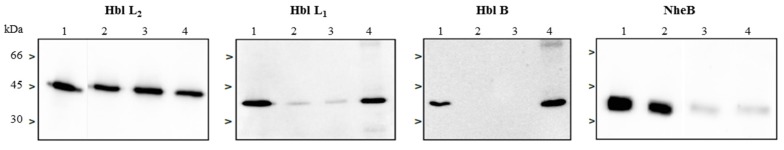
Western blot analyses of Hbl B-specific IAC fractions of MHI 1532 supernatant: 1, supernatant of strain MHI 1532; 2, sample flow-through; 3, wash fraction; and 4, elution fraction. Toxin components were detected using the following mAbs: 1H9 (Hbl L2), 1E9 (Hbl L1), 1B8 (Hbl B), and 1E11 (NheB) ([20,33] and this study).

In an independent approach, MHI 1532 supernatant was purified via IAC using the Hbl B-specific mAb 1B8 [29] equivalently to the generation of the immunogen. Single fractions were collected and tested in sandwich EIAs. Results of three independent approaches are summarized in Table 2. Hbl B and L_1_ bound with high capacity to the IAC column, less than 5% of these toxin components were detected in sample flow-through and wash fraction. The detectable proportion of Hbl B and L_1_ in the elution fraction averaged at 187% and 180% compared to the MHI 1532 supernatant. The recovery rate of Hbl L_2_ in the elution fraction averaged at only 29%, while 51% were detected in the sample flow-through and 20% in the wash fraction. A sandwich EIA against the toxin component NheB was performed to exclude unspecific binding of toxin components to the immunosorbent. Indeed, 97% of NheB were detected in the sample flow-through. As a control, the identical experiment was performed using the Hbl L_2_-specific mAb 1H9 coupled to the CnBr-activated sepharose 4B matrix. Ninety percent of Hbl L_2_ were retrieved in the elution fraction, whereas only 2% and 1% of Hbl L_1_ and B were detected, respectively (Table 2). Corresponding Western blot analyses confirmed the foregoing results (Figure 7). Altogether, Hbl B as well as L_2_ and L_1_ could be detected in the elution fraction of the Hbl B-specific 1B8-IAC and prove the pronounced complex formation between the single Hbl components in natural supernatants. 

## 3. Discussion

With the construction of an *nheABC* deletion mutant and the establishment of a system for overexpression and purification of active recombinant Hbl components in *E. coli*, the first important steps towards clarification of the molecular mode-of-action of Hbl have been made. Earlier studies had been laborious and not fully conclusive, as the second three-component enterotoxin Nhe is always present in crude *B. cereus* culture supernatants [28,29]. Furthermore, Hbl was purified in these early studies via anion exchange chromatography, including the possibility that the resulting preparations might have contained trace contaminations of Nhe [10,23,30]. As Nhe dominates toxicity studies, another approach to assess the peculiar Hbl activity was to remove NheB via subtractive immunoaffinity chromatography (IAC) and attribute the remaining toxic activity to Hbl [33]. By using rHbl components in the present study, no other *B. cereus* toxin is involved and the single Hbl components can be applied in defined concentrations and proportions. The three rHbl components produced in *E. coli* are functionally active and cytotoxic, but it must be admitted that cytotoxic activity is reduced compared to natural Hbl appearing in *B. cereus* culture supernatants (refer to Figure 1C,D). 

Based on these reactive proteins and a broad range of Hbl-specific mAbs, complex formation between the single Hbl components could be detected for the first time. Via Dot blots, hybrid sandwich EIAs and immunoaffinity chromatography, Hbl L_1_-B and Hbl L_1_-L_2_ complex formation could be shown both for the recombinant Hbl proteins as well as in natural *B. cereus* culture supernatants (refer to Figure 3, Figure 4 and Figure 6). Particularly, the L_1_ and B components seem to be highly complexed. The only, comparably weak interaction between Hbl B and L_2_ was observed in SPR measurements, when rHbl B was immobilized and high concentrations of rHbl L_2_ were added (refer to Figure 5C). Apart from that, SPR measurements confirmed the results obtained in hybrid sandwich EIAs, indicating that there is an interaction and complex formation between Hbl L_1_ and L_2_ as well as L_1_ and B. This Hbl complex formation also explains why all three Hbl components were detected in the elution fraction of Hbl B-specific IAC from strain MHI 1532 (Table 2 and Figure 7), which was initially used for immunogen preparation, and why mAbs specific for all three Hbl components (Table S1) were generated. Thus, it can be postulated that in natural *B. cereus* culture supernatants Hbl B is to a large extent bound in complexes. Further on, these complexes are characterized by K_D_ values of 4.7 × 10^−7^ M (L_1_-B) and 3.4 × 10^−6^ M (B-L_2_) (Figure 5). These are relatively low constants compared to the situation found for the NheB-C complexes, for which a value of 4.8 × 10^−10^ M was found [34]. This might be the reason why single Hbl components were obtained from *B. cereus* culture supernatants with the applied classical chromatographic approach in the early studies [23,30].

According to sequence homologies between the two three-component enterotoxin complexes of *B. cereus*, Hbl L_2_ correlates with NheA, Hbl L_1_ with NheB and Hbl B with NheC [31,35]. Thus, the Hbl L_1_-B complexes found in this study would correspond to NheB–NheC complexes, which have already been well-described. It was shown that a defined level of NheB–NheC complexes as well as a sufficient amount of free NheB is necessary for efficient cell binding and toxicity [21,34]. Based on these data, a detailed model on the mode-of-action of Nhe could be developed [36]. Assuming that Hbl L_1_ is equivalent to NheB and Hbl B to NheC, it can be speculated that the Hbl L_1_-B complexes alone might be able to bind to the target cells and form a kind of “pro-pore” [34]. Analogously to NheA, Hbl L_2_ would be the third component which, presumably after undergoing conformational changes, binds to the complex and completes the pore. However, the comparably low K_D_ values of the complexes found in this study, as well as the proposed binding order of the single components B-L_1_-L_2_ [19] oppose this theory.

Interestingly, in our study some of the newly generated mAbs against Hbl B enhanced Hbl toxicity towards Vero cells (refer to Figure 2A). By using purified rHbl B and flow cytometry analyses, an increased binding of Hbl B to Vero cells in the presence of the Hbl B-specific mAb 1D7 was found (Figure 2B and Table 1). Various studies describe enhancing effects of virus-specific mAbs, known as antibody-dependent-enhancement (ADE) [37,38]. ADE has been described for Dengue fever [39], HIV [40], Feline Infectious Peritonitis [41] and Ebola [42]. In 2004, ADE was first observed regarding the lethal toxin of *B. anthracis* [43]. The authors postulated stabilization of PA (protective antigen) binding to the cell surface via interaction of mAbs with macrophage Fcγ receptors. In contrast to this, involvement of Fc receptors can be excluded in our study, as they do not occur on the surface of Vero cells. Thus, the reason for the increased rHbl B binding remains unclear. One can speculate that through the two binding sites of the mAb, an antibody-mediated cross-linking of rHbl B is induced, which might promote the binding of rHbl B to the cell surface. It is further possible that binding of the mAb results in a change of the tertiary structure of rHbl B enabling a better binding to the target cells.

Besides these toxicity-enhancing mAbs, also mAbs with neutralizing properties were also found. This is particularly true for the mAbs reactive with Hbl L_2_ (refer to Table S1 and Figure 2A). However, these mAbs neutralize Hbl toxicity only by approximately 60%, in contrast to an earlier study, in which an NheB-specific mAb (1E11) was generated that neutralizes Nhe-dependent toxicity almost completely [20]. The partial neutralization of Hbl L_2_-specific 1H9 and 1D8 indicates that both mAbs interact with regions of the toxin which are important, but not mandatory for toxic activity. Further studies are necessary to determine whether these mAbs hinder the proposed conformational change of the Hbl components during pore formation [26] or the putative attachment of Hbl L_2_ to cell-bound Hbl B/L_1_. It might also be possible that the mAbs are not able to access Hbl L_2_, as it is partially bound in complexes with the other components, as shown in this study. 

The demonstration of Hbl complex formation in the current study is a first step to characterize its mode-of-action in detail. Further studies are in progress to elucidate the epitope specificity of the mAbs to get new insights into the interaction of the single components. Cell culture studies will follow to investigate the impact of complex formation on the functionality and toxic activity of Hbl. Thus it can be elucidated if the complexes either support or hinder pore formation and if—next to Nhe—another example can be found for the so far unique, permeable “pro-pores”, or if Hbl conceals a different mechanism of hetero-oligomerization leading to pore formation.

## 4. Materials and Methods

### 4.1. Ethics Statement

Immunizations of mice for generating monoclonal antibodies were conducted in compliance with the German Law for Protection of Animals. Study permission was obtained by the Government of Upper Bavaria (permit number 55.2-1-54-2532.6-2-12).

### 4.2. Cell Lines and Culture Conditions

Vero cells were obtained from ECACC (European Collection of Cell Cultures) and cultured in 80 cm^2^ culture flasks in a humidified incubator at 37 °C and 7% CO_2_ in medium recommended by the supplier. 

### 4.3. Bacterial Strains and Culture Conditions

In this study, the *B. cereus* strains F837/76 (DSM 4222), B4ac (DSM 4384), MHI 1532 (producing high amounts of Hbl toxin) and F837/76 ∆*nheABC* (producing Hbl, but no Nhe toxin) were used. For collection of toxin-rich supernatants, cells were grown in CGY medium with 1% glucose and treated as previously described [33]. Recombinant Hbl proteins were overexpressed in the *E. coli* strain BL21 (DE3), which was grown in LB medium containing 100 μg/mL ampicillin.

### 4.4. Construction of the nheABC Deletion Mutant

The *nheABC* deletion mutant was constructed as described previously with minor modifications [44]. Briefly, for the in-frame deletion of the *nheABC* gene cluster, an 808 bp upstream fragment of bcf_09260 (*nheA*) and an 813 bp downstream fragment of bcf_09270 (*nheC*) were amplified by PCR using the primer pairs nheA-BamHI/nheA-Spc (CGGGATCCCGAGTTACTGTCGTTATACC; CGTTAGCGTTTAAGTACATCCCCTGTAATTAAAGTCTTTTTCAC) and nheC-Spc/nheC-EcoRI (GCGTCCTCTTGTGAAATTAGAGGATTATACAGAAAAATTACATGAAG; CGGAATTCCTCCATTATACGGTTCACTCG). To allow the selection of positive clones, the spectinomycin-resistance cassette from the TOPO/Spc plasmid was amplified with oligonucleotides For_Spc-K/Rev_Spc-K (GGATGTACTTAAACGCTAACG/CTCTAATTTCACAAGAGGACGC) and inserted between upstream and downstream fragments using ligation-independent cloning (LIC) of PCR products [45]. Following the next PCR using the primer pair nheA-BamHI/nheC-EcoRI and the LIC construct as a template, the resulting fragment was cloned into the multiple cloning site of the conjugative suicide vector pAT113 via BamHI and EcoRI, giving rise to pAT113-SpcΔ*nheABC*. *E. coli* JM83/pRK24 was transformed with pAT113-SpcΔ*nheABC* and the resulting strain was used for transconjugal transfer into *B. cereus* F837/76. Conjugation was carried out as described [46]. Transconjugants were screened for spectinomycin resistance and erythromycin sensitivity. Gene cluster deletion and integration of the resistance cassette resulting from a double-crossover recombination event were confirmed by PCR and sequencing.

### 4.5. Cloning of Recombinant Hbl (rHbl) Components

The genes encoding Hbl L_2_ (*hblC*; bcf_15295), Hbl L_1_ (*hblD*; bcf_15290) and Hbl B (*hblA*; bcf_15285) were amplified using the primer pairs HblL_2_-fw-KpnI (ATATGGTACCCCAAGCAGAAACTCAACAAGAAA) and HblL_2_-rev-NcoI (ATATCCATGGTCAAAATTTATACACTTGTTCTTC), HblL_1_-fw-SacII (ATATCCGCGGATGGCACAAGAAACGACCGCTCAAG) and HblL_1_-rev-NcoI (ATATCCATGGGCCTCCTGTTTAAAAGCAATATC) and HblB-fw-KpnI (ATATGGTACCCGCAAGTGAAATTGAACAAACGAAC) and HblB-rev-NcoI (ATATCCATGGCTATTTTTGTGGAGTAACAGTTTCTAC), respectively. Chromosomal DNA from strain F837/76 was used as template. With the chosen primers, genes were amplified lacking the sequences for the N-terminal signal peptides for secretion [47,48]. *hblD* was cloned into pASK-IBA3plus (iba lifesciences), adding a C-terminal strep-tag to the recombinant Hbl L_1_ protein. *hblC* and *hblA* were cloned into pASK-IBA5plus (iba lifesciences), resulting in addition of N-terminal strep-tags. Sequencing was performed using the primers pASK-IBA-seq-fw (CACTCCCTATCAGTGATAG) and pASK-IBA-seq-rev (GCACAATGTGCGCCAT).

### 4.6. Overexpression of rHbl Components

For overexpression of the rHbl components the *E. coli* strain BL21 (DE3) was used. Fifty millimetres LB medium with 100 μg/mL ampicillin were inoculated to an OD_600_ of 0.15. Cells were grown at 37 °C to an OD_600_ of 0.8–0.9. After overexpression was induced by adding 1 μg/mL doxycycline, cells were grown for further four hours and then harvested by centrifugation.

### 4.7. Purification of rHbl Components

The *E. coli* cell pellet was dissolved in 15 mL resuspension buffer (100 mM Tris, 500 mM NaCl, 1 mM EDTA, pH 8) containing protease inhibitor complete (Roche) and 100 μg/mL lysozyme. Cells were subsequently disrupted in an ultrasonic bath for 2 × 15 min on ice. Cell debris was removed by centrifugation for 30 min at 14,000× *g* and 4 °C. The remaining cell extract was filtered through a 0.2 nm filter. All strep-tagged Hbl components were purified via a 0.5 mL Strep-Tactin^®^-Sepharose^®^ matrix (iba lifesciences) in a Poly-Prep chromatography column (Biorad) according to the protocol provided by the supplier. Proteins were eluted in buffer containing 100 mM Tris, 500 mM NaCl, 1 mM EDTA and 2.5 mM D-desthiobiotin, pH 8. Protein concentrations were determined after SDS-PAGE by staining with Sypro Ruby in comparison to a BSA concentration standard. 

### 4.8. Production of Monoclonal Antibodies (mAbs)

For the preparation of the immunogen, toxin was purified via immunoaffinity chromatography (IAC) from a total of 300 mL supernatant of *B. cereus* strain MHI 1532 grown in CGY medium. For this purpose, the previously established Hbl B-specific mAb 1B8 (10 mg) [29] was coupled to 1 g CNBr-activated sepharose 4B. The column was washed with 20 mL PBS before the toxin-containing culture supernatant was applied. After a washing step with 20 mL PBS, the toxin was eluted by addition of 16 mL glycine/HCl solution (pH 2.5). Subsequently, the eluate was neutralized with 1 M Tris (pH 7.0) and dialyzed three times overnight at 4 °C against PBS. Five 12-week-old female mice (BALB/c and BALB/c × [NZW × NZB]) were immunized with this preparation. Each mouse received 15 μg purified toxin (immunization and first booster injection after 9 weeks) and another 12 μg (second booster injection after 15 weeks) emulsified in Sigma adjuvant. Three days before cell fusion each mouse received a final injection of 20 μg toxin diluted in 0.9% NaCl-solution. Cell fusion experiments, establishment of hybridomas, mass production and antibody purification were performed as described previously [20,32]. 

### 4.9. SDS-PAGE, Sypro Staining and Immunoblotting

SDS-PAGE analyses were carried out on a PhastGel gradient (10 to 15%) minigel system (GE Healthcare, Munich, Germany). For Sypro staining, proteins were fixed on the gel for 2 × 30 min in 50% MeOH and 7% acetic acid. The gel was then incubated with 2 mL Sypro Ruby protein stain overnight at room temperature. After 30 min washing in 10% MeOH and 7% acetic acid and additional washing for 10 min in H2O, fluorescence signals were detected on a Kodak imager (Eastman Kodak Company, Rochester, NY, USA).

For immunoblotting, proteins were blotted to a PVDF-P membrane (Millipore, Billerica, MA, USA), blocked in 3% casein-PBS and incubated with 2 μg/mL mAbs 8B12 (Hbl L2) [32], 1E9 (Hbl L1) [29], 1B8 (Hbl B) [29], 1E11 or 2B11 (NheB) [20], 1A8 (NheA) [20], 3D6 (NheC) [21], mAb 1H9 (this study), or the strep-specific StrepMAB-Classic (iba lifesciences) for 1 h at room temperature. After 3 washing steps in PBS with 0.1% Tween 20, a 1:2000 dilution of rabbit anti-mouse-horseradish peroxidase conjugate (Dako) was used as secondary antibody. After three further washing steps in PBS with 0.1% Tween 20 and two in PBS, Super Signal Western Femto Maximum Sensitivity Substrate (Pierce) was applied. Chemiluminescence signals were detected on a Kodak imager (Eastman Kodak).

### 4.10. Labelling of mAbs

For use as detection antibodies in the sandwich EIAs, mAbs were coupled to activated peroxidase (HRP) according to the instructions of the manufacturer (Roche). The resulting conjugate was stabilized with 1% BSA and StabilZyme^®^ HRP Conjugate Stabilizer (SurModics) and conserved with 0.01% Thimerosal. 

For flow cytometric analyses, mAb 1G8 was labelled with Alexa Fluor^®^ 488 (Thermo Scientific, Waltham, MA, USA). For that, 2 mg 1G8 (in 1 mL PBS) were mixed with 200 μL Alexa Fluor^®^ 488. The mixture was stirred gently for 1 h at room temperature in the dark. To remove unbound Alexa dye, 10 mL PBS were added and the material was transferred to a Amicon^®^ Ultra-4 centrifugal filter unit (Millipore) and centrifuged for 20 min (3000 rpm, 4 °C). The labelled mAb was washed with another 10 mL PBS by repeating the centrifugation step, and afterwards resuspended in PBS. NaN_3_ (0.1% ) was added for conservation and 1% BSA for stabilization.

### 4.11. Flow Cytometry

For flow cytometry analyses, Vero cells were adjusted to 1 × 10^6^ cells in 1 mL EC buffer (140 mM NaCl, 15 mM HEPES, 1 mM MgCl_2_, 1 mM CaCl_2_, 10 mM glucose pH 7.2). Then 6.5 pmol/mL rHbl B and, if applicable, mAbs were added (ratio rHbl B:mAb = 1:1). The mixture was incubated for 1 h at 37 °C under moderate agitation. Then, 2 mL 1% BSA-PBS were added and cells were centrifuged for 5 min at 800 rpm. Cells were washed again in 2 mL 1% BSA-PBS. When rHbl B and mAb 1D7 were applied successively, the incubation step at 37 °C and the washing step were repeated. For detection of cell-bound rHbl B, the samples were incubated with 3 μg/mL Hbl B-specific Alexa Fluor^®^ 488-labelled mAb 1G8 for 1 h at 4 °C. After additional washing, samples were resuspended in 500 μL 1% BSA-PBS and subsequently analysed in a FACS Calibur using the CellQuestPro software (BD Bioscience). 

### 4.12. Enzyme Immunoassays (EIAs) 

Indirect and sandwich enzyme immunoassays were performed as described before [20,28,32,49].

For detection of rHbl components, established mAbs were used (1A12 and 8B12 [32] for Hbl L_2_, 1E9 [29] for Hbl L_1_ and 1B8 [29] for Hbl B), as well as the ones newly generated in this study (Table S1). To determine relative affinities of the newly generated mAbs, *B. cereus* culture supernatants and rHbl components were used in indirect EIAs. For that, dilution series of the antigens were applied, followed by the cell culture supernatants (constantly 1:20 in PBS). The relative affinity corresponds with the dilution that results in an absorbance value of 1.0. The productivity of the hybridoma cell lines was also determined in indirect EIAs. Supernatant of *B. cereus* MHI 1532 was applied (constantly 1:10 in PBS), followed by dilution series of the cell culture supernatants. 

rHbl complex formation was investigated using indirect and sandwich EIAs. In the indirect assay, the microtiter plate was coated with a serial dilution of rHbl L_1_ (120–0 pmol/mL) overnight. After washing, the second rHbl component was applied in constant concentration (60 pmol/mL) for 1 h. After blocking for 30 min with 3% sodium-caseinate in PBS, HblB-specific mAb 1B8 [29] and Hbl L_2_-specific mAb 1H9 (this study) (2 μg/mL in PBS) were applied, respectively. After additional washing, rabbit-anti-mouse-HRP conjugate (1:2000 in 1% sodium-caseinate in PBS) was applied for detection. In the sandwich assay, the microtiter plate was coated with 10 μg/mL mAbs 1E9 [29] or 1G8 (this study), both Hbl L_1_-specific, overnight. While the plate was blocked for 30 min with 3% sodium-caseinate in PBS, a mixture of rHbl components (L_1_+B or L_1_+L_2_; 1.5 pmol/μL each, ratios 1:1, 5:1, 1:5, 10:1 or 1:10) was incubated at RT. The mixtures were applied to the microtiter plate as serial dilution from 75 to 0 pmol/mL. After washing, a specific mAb conjugate was applied for detection (1:2000 in 1% sodium-caseinate in PBS; 1B8-HRP [29] against Hbl B or 1H9-HRP (this study) against Hbl L_2_). Analogously to the detection of the rHbl components, sandwich EIAs were used to detect Hbl complexes in the supernatant of *B. cereus* strain F837/76, which was applied to the microtiter plates as serial dilution. One-site-binding curves were applied to depict the absorption values compared to the sample dilutions.

### 4.13. Dot Blot 

One hundred microlitres per dot of the first purified rHbl component, diluted in PBS, were applied to a PVDF membrane (Immobilon-P, Millipore, USA) as dilution series from 480 to 3.75 pmol. The membrane was removed from the dotting chamber and blocked with 3% sodium-caseinate-PBS overnight. Subsequently, the second purified rHbl component was applied (30 pmol in PBS) and overlaid on the dots for 1 h. The membrane was washed for 3 × 10 min in PBS containing 0.1% Tween 20. After that, Hbl-specific mAbs were applied (1B8 against Hbl B [29], 1H9 against Hbl L_2_ (this study); 1E9 [29] and 1G8 (this study) against Hbl L_1_; 3 μg/mL in 3% sodium-caseinate-PBS with 0.025% Tween 20) and overlaid on the dots for 1 h. The membrane was again washed for 3 × 10 min in PBS containing 0.1% Tween 20 before anti-mouse-IgG-alkaline-phosphatase-conjugate (Sigma) was applied (1:10.000 in 3% sodium-caseinate-PBS with 0.025% Tween 20) for 1 h. Again, the membrane was washed for 3 × 10 min in PBS containing 0.1% Tween 20 and for 2 × 10 min in PBS before signals were detected using NBT/BCIP solution (Roche).

### 4.14. Surface Plasmon Resonance (SPR)

Binding measurements were performed on a BIACORE 3000 instrument (Biacore Inc., Piscataway, NJ, USA). In a first experiment, rHbl L_1_ was coupled and rHbl L_2_ and B were used as ligands. For that, L_1_ was diluted to a final concentration of 416 nM in 10 mM sodium acetate, pH 4.8, and chemically immobilized (amine coupling, 560 RU bound) onto a CM5 sensor chip (Biacore Inc.). The L_2_ and B protein samples were diluted in running buffer (PBS, 1 mM DTT and 0.005% Tween 20) and injected over the sensor chip surface at 30 μL/min at 20 °C. The samples were injected onto the sensor chip from the lowest to the highest concentration with injection of 250 nM ligand in duplicate within each experiment. L_2_ and B protein samples were measured three and four times, respectively. 

For the second experiment, rHbl B was diluted to a final concentration of 350 nM in 10 mM sodium acetate, pH 4.0. 500 RU were coupled onto a CM5 sensor chip. rHbl L_2_ was diluted in the same running buffer as above and injected over the sensor chip surface at 30 μL/min at 20 °C. The experiment was repeated four times. Background subtraction was done using an unmodified sensor chip surface. Data were analysed using BIAevaluation program (Biacore Inc.). For each measurement the equilibrium dissociation constant (K_D_) was calculated from steady-state measurement. The K_DS_ from three or four experiments (see above) were used to calculate mean values and standard deviations. Despite long injection times the measurement of L_1_ + B did not reach full binding saturation for lower protein concentrations. Therefore, the derived K_D_ value is likely to be slightly underestimated.

### 4.15. Cytotoxicity Assays

Stock solutions (1.5 pmol/μL) of the rHbl components were used for toxicity assays on Vero cells. They were pre-mixed in appropriate ratios and added in 1:40 dilutions to the cells. Propidium iodide (PI) influx tests were performed as described before [33,49]. WST-1 bioassays were carried out as previously described [20,32,49]. For neutralization assays, 10 μg/well purified mAbs were applied constantly with a serial dilution (starting 1:20) of culture supernatant of *B. cereus* strain F837/76 ∆*nheABC*.

## Figures and Tables

**Table 1:** Flow cytometry of Vero cells treated with rHbl B. Vero cells (1 × 10^6^) were incubated for 1 h with rHbl B (6.5 pmol/mL) and simultaneously with mAbs 1D7 (Hbl B-specific), 1D12 (Hbl B-specific) or 12D12 (Hbl B/L_1_-specific) (ratio mAb:rHbl B = 1:1). Fluorescence was detected via Hbl B-specific Alexa Fluor^®^ 488-labelled mAb 1G8 (FL1, fluorescence at 488 nm).

Sample	FL1-Positive (%)
negative controls:	
-	0.69 ± 0.29
1D7	0.96 ± 0.12
1D12	0.68 ± 0.03
12D12	0.53 ± 0.06
rHbl B and mAbs co-incubated:	
rHbl B	41.95 ± 6.3
rHbl B + 1D7	96.37 ± 4.34
rHbl B + 1D12	43.99 ± 6.42
rHbl B + 12D12	50.81 ± 4.11
rHbl B and mAb 1D7 consecutively:	
rHbl B, 1D7	32.76 ± 24.61
isotype controls:	
rHbl B + IgG1	58.37 ± 7.33
rHbl B + IgG2a	46.36 ± 1.34

**Table 2:** Proportion of Hbl components L_2_, L_1_ and B in sample flow-through, wash and elution fraction after IAC purification of *B. cereus* MHI 1532 supernatant. Hbl B-specific mAb 1B8 [29] and Hbl L_2_-specific mAb 1H9 (this study) were used for IAC. Hbl components were determined in sandwich EIAs: Hbl L_2_: mAbs 1A12 [32]/1H9-HRP (this study); Hbl L_1_: mAbs 1E9 [29]/1G8-HRP (this study); Hbl B: mAbs 1D12 (this study)/1B8-HRP [29]. NheB was also measured as a control (mAbs 2B11/1E11-HRP) [20]. EIA titres were multiplied with the respective sample volume resulting in units. The respective units determined in MHI 1532 supernatant were set to 100%.

**Hbl B-Specific IAC (1B8)**			
	**Sample Flow-Through**	**Wash Fraction**	**Elution Fraction**
Hbl L_2_	51%	20%	29%
Hbl L_1_	<5%	<5%	180%
Hbl B	<5%	<5%	187%
NheB	97%	-	3%
**Hbl L_2_-Specific IAC (1H9)**			
	**Sample Flow-Through**	**Wash Fraction**	**Elution Fraction**
Hbl L_2_	9%	1%	90%
Hbl L_1_	55%	4.5%	2%
Hbl B	110%	10%	1%

## Supplementary Materials

The following are available online at www.mdpi.com/2072-6651/9/9/288/s1. **Table S1:** Characteristics of the established hybridoma cell lines and mAbs, **Table S2:** Statistics of the curves fitted to the EIA and SPR experiments, **Figure S1:** Detection of Hbl complex formation via Dot blot.
