# Supplementary material for: Evidence for Complex Formation of the Bacillus cereus Haemolysin BL Components in Solution
Source: Toxins (Basel). 2017 Sep 16;9(9):288. doi: 10.3390/toxins9090288 (PMC5618221; doi:10.3390/toxins9090288)
Supplement: Supplementary file 1 [file toxins-09-00288-s001.pdf]

Supplementary Materials: Evidence for Complex Formation of the *Bacillus cereus* Haemolysin BL Components in Solution

Franziska Tausch, Richard Dietrich, Kristina Schauer, Robert Janowski, Dierk Niessing, Erwin Märtlbauer and Nadja Jessberger

Table S1. Characteristics of the established hybridoma cell lines and mAbs.

fusion	cell line	Ig subtype	target	productivity (reciprocal antibody titre)	relative affinity[a] B4ac supernatant	rHbl components
I	1B3	IgG_1	Hbl B	7000	60	800
I	1C10	IgG_1	Hbl B	15400	140	760
I	1C11	IgG_{2b}	Hbl B	16200	50	450
I	1D12	IgG_{2a}	Hbl B	13900	100	480
I	1E7	IgG_1	Hbl B	6300	120	620
I	1E8	-	Hbl B	70	30	100
I	1K9	IgG_1	Hbl B	6000	80	490
I	2G4	IgG_1	Hbl B	12700	120	690
I	2H7	IgM	Hbl B	3400	80	540
I	11A5	IgG_1	Hbl B	9000	170	900
I	1G8	IgG_1	Hbl B / Hbl L1	850	130	900 / 610
I	11H5	IgG_1	Hbl B / Hbl L1	20500	230	410 / 680
I	12D9	IgG_1	Hbl B / Hbl L1	>21900	340	580 / 890
I	12D12	IgG_{2a}	Hbl B / Hbl L1	15900	70	180 / 440
II	1B10	IgG_1	Hbl B	580	30	<10
II	1D7	IgG_1	Hbl B	6300	110	270
II	1D9	-	Hbl B	250	60	310
II	1E1	IgG_{2b}	Hbl B	6400	40	330
II	1E4	-	Hbl B	2900	60	370
II	1E5	-	Hbl B	6700	60	200
II	1E7	-	Hbl B	3500	50	250
II	1F6	IgG_1	Hbl B	1800	30	370
II	2H3	IgG_1	Hbl B	720	20	390
II	1D8	IgG_{2a}	Hbl L2	4800	580	4700
III	1A10	IgG_1	Hbl B	80	20	<10
III	1B10	-	Hbl B	600	80	130
III	1C9	-	Hbl B	13700	40	360
III	1C12	IgG_{2b}	Hbl B	1400	60	140
III	1E2	IgG_1	Hbl B	>21900	60	780
III	1E10	IgG_1	Hbl B	>21900	80	390
III	1F9	IgG_1	Hbl B	4000	60	270
III	1H1	-	Hbl B	17900	80	550
III	2G10	IgG_1	Hbl B	18400	90	580
III	2H10	IgG_1	Hbl B	>21900	20	230
III	1H9	IgG_1	Hbl L2	820	1500	8400

[a] : Reciprocal titre in indirect EIAs.

- : No distinct Ig isotyping results.

Table S2. Statistics of the curves fitted to the EIA and SPR experiments. Analyses of EIA data were performed using GraphPad Prism Version 5.04 for Windows, GraphPad Software, San Diego California USA, www.graphpad.com. Correlation coefficients and the type of equation used are shown. For the SPR experiments (Figure 5) a one-site binding model was used and the statistical value chi^2 was calculated with BIAevaluation program.

graph		equation type non-linear regression	R^2
Figure 3B			
L_1 - B - mAb 1B8		saturation binding curve	0.9723
L_1 - L_2 - mAb 1H9		saturation binding curve	0.9505
Figure 4A			
mAb 1E9 - L_1+B - mAb 1B8-HRP	L_1+B	saturation binding curve	0.9997
	L_1	saturation binding curve	0.8337
	B	saturation binding curve	0.6166
mAb 1G8 - L_1+L_2 - mAb 1H9-HRP	L_1+L_2	saturation binding curve	0.9909
	L_1	saturation binding curve	0.9424
	L_2	saturation binding curve	0.4649
Figure 4B			
mAb 1E9 - L_1+B - mAb 1B8-HRP	1:1	one phase decay	0.9707
	5:1	one phase decay	0.9883
	10:1	one phase decay	0.9750
	1:5	one phase decay	0.9359
	1:10	one phase decay	0.9478
mAb 1G8 - L_1+L_2 - mAb 1H9-HRP	1:1	one phase decay	0.9853
	5:1	one phase decay	0.9859
	10:1	one phase decay	0.9814
	1:5	one phase decay	0.9835
	1:10	one phase decay	0.9874
Figure 5			chi^2
A. L_1 + L_2		one-site binding model	8.06
B. L_1 + B		one-site binding model	3.49
C. B + L_2		one-site binding model	4.48
Figure 6			
A. mAb 1E9 - supernatant - mAb 1B8-HRP		one phase decay	0.9842
B. mAb 1G8 - supernatant - mAb 1H9-HRP		one phase decay	0.9526
C. mAb 1H9 - supernatant - mAb 1G8-HRP		one phase decay	0.9221

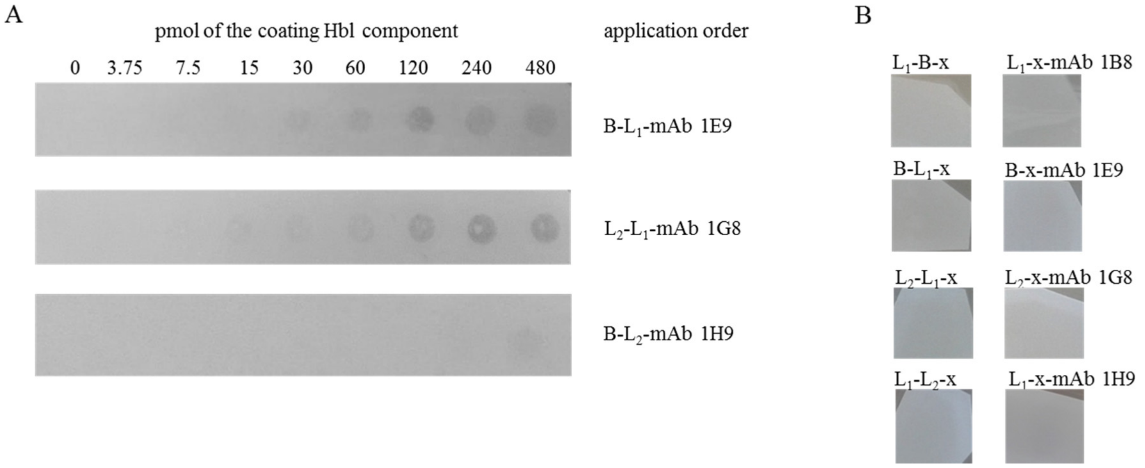

Figure S1. Detection of Hbl complex formation via Dot blot. A. The first rHbl component was applied to the PVDF membrane in rising concentrations (3.75 - 480 pmol). After blocking, the membrane was incubated in PBS with the second component (30 pmol). Proteins were detected using the specific mAbs 1E9 (Hbl L_1) [1], 1G8 (Hbl L_1) (this study) and 1H9 (Hbl L_2) (this study) and a goat anti mouse-alkaline phosphatase conjugate. **B.** Negative controls showed the specificity of the reaction.

References

1. Wehrle, E.; Moravek, M.; Dietrich, R.; Bürk, C.; Didier, A.; Märtlbauer, E. Comparison of multiplex PCR, enzyme immunoassay and cell culture methods for the detection of enterotoxinogenic *Bacillus cereus*. *J. Microbiol. Methods* **2009**, *78*, 265–270. doi:10.1016/j.mimet.2009.06.013.
